# Supplementary figures and images for: Evaluation of film stimuli for the assessment of social-emotional processing: a pilot study
Source: PeerJ. 2022 Nov 23;10:e14160. doi: 10.7717/peerj.14160 (PMC9700451; doi:10.7717/peerj.14160)

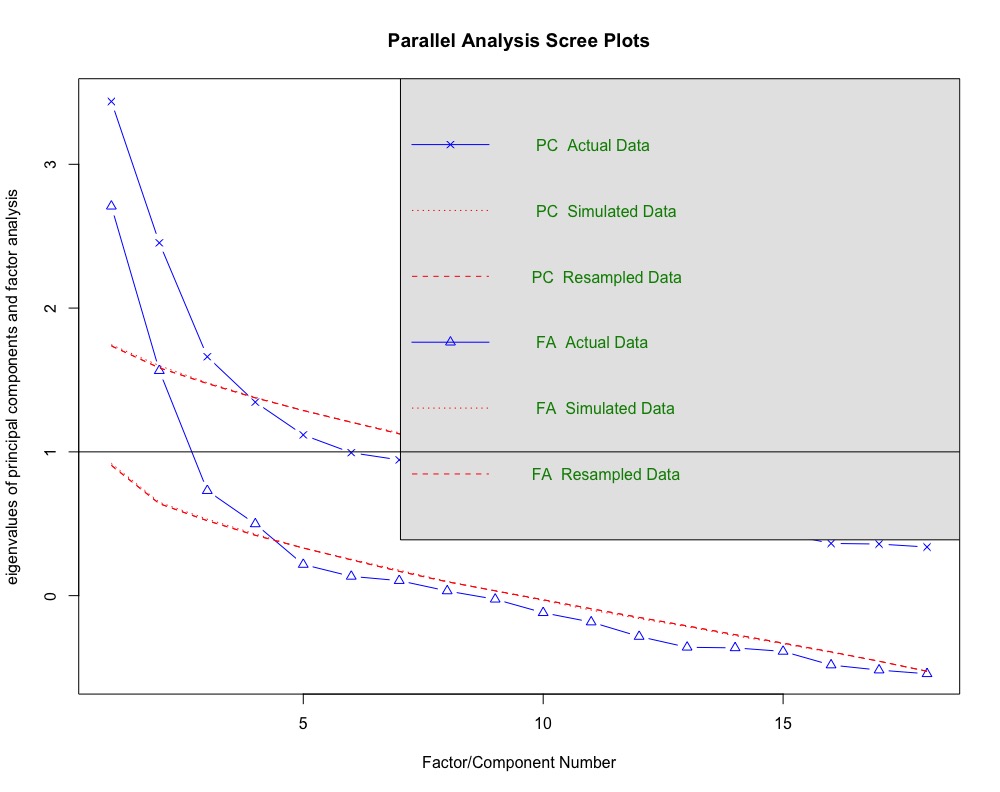

Supplement: Supplemental Information 1 [file peerj-10-14160-s001.jpeg]

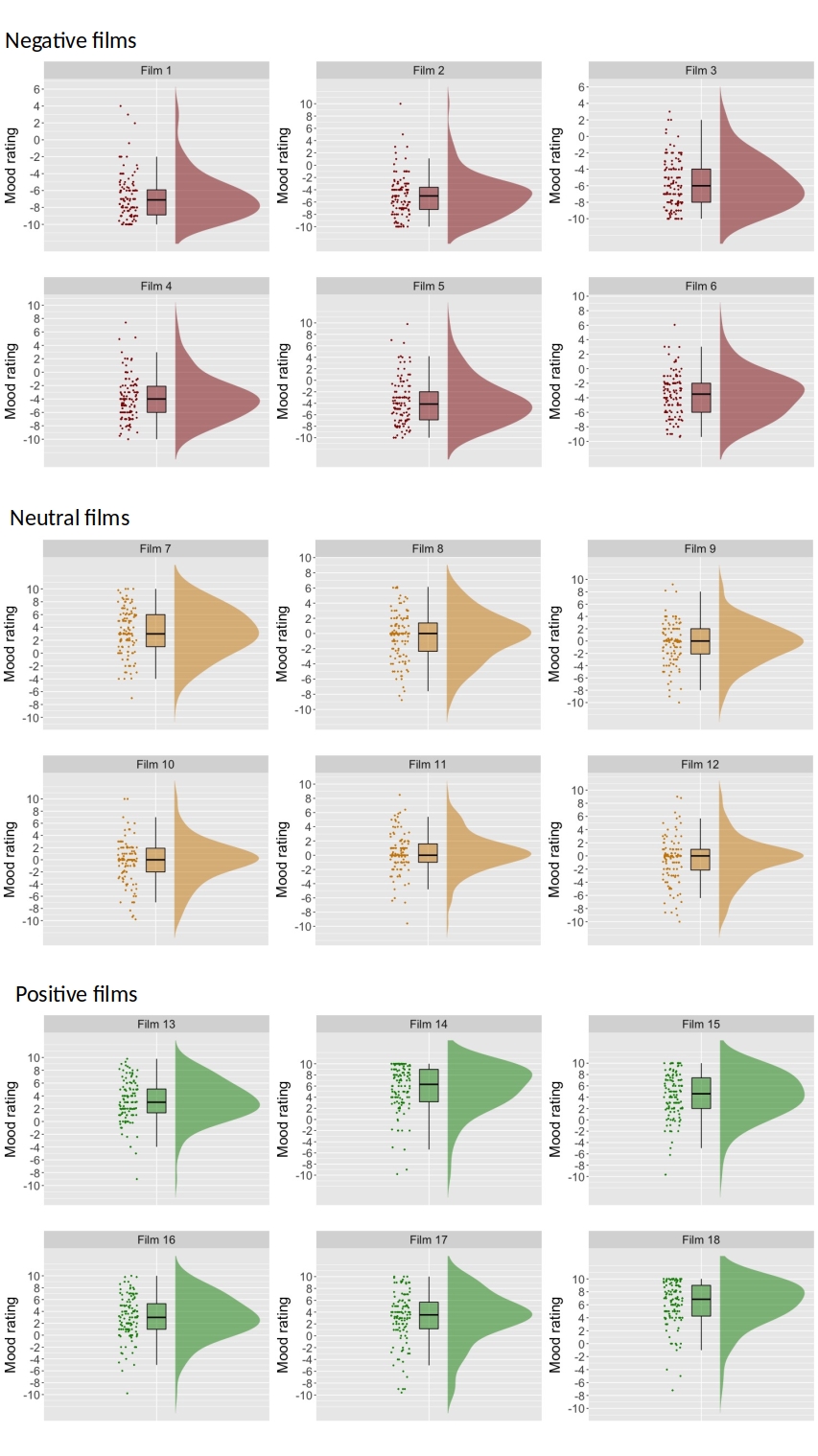

Supplement: Supplemental Information 2 — The dots represent individual data points. The box plot summarises minimum, first quartile, median, third quartile, and maximum. The half violin plots shows the density of the data. [file peerj-10-14160-s002.jpg]

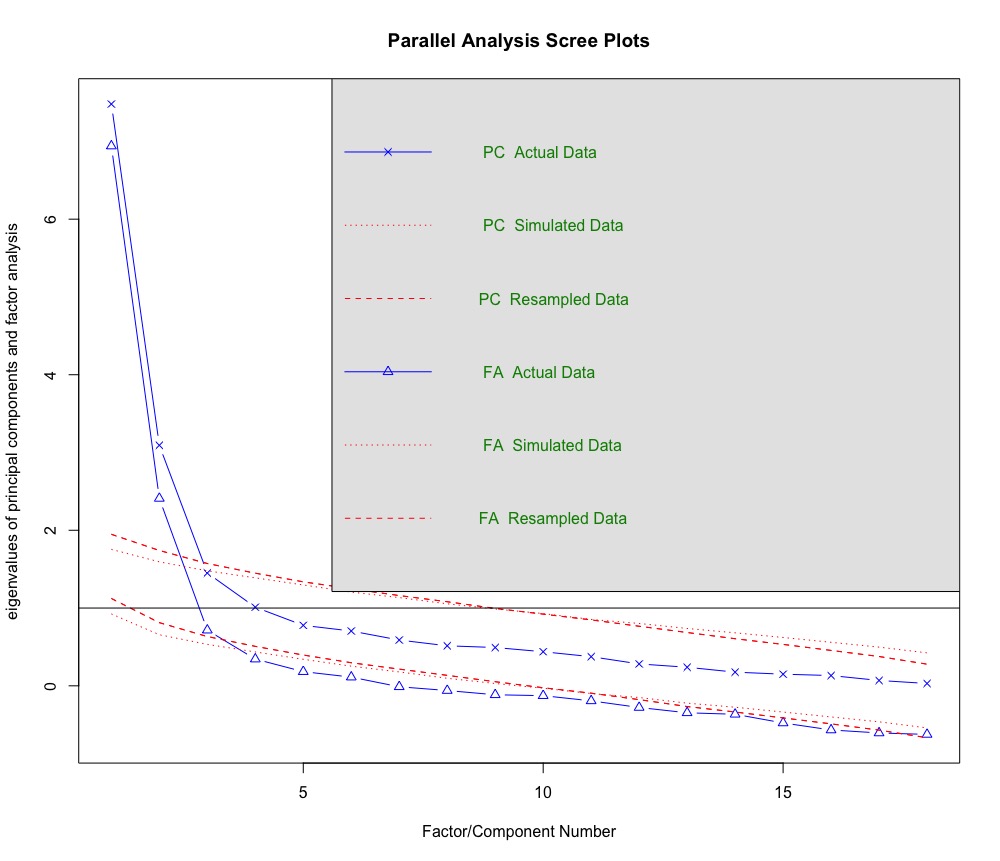

Supplement: Supplemental Information 3 [file peerj-10-14160-s003.jpeg]

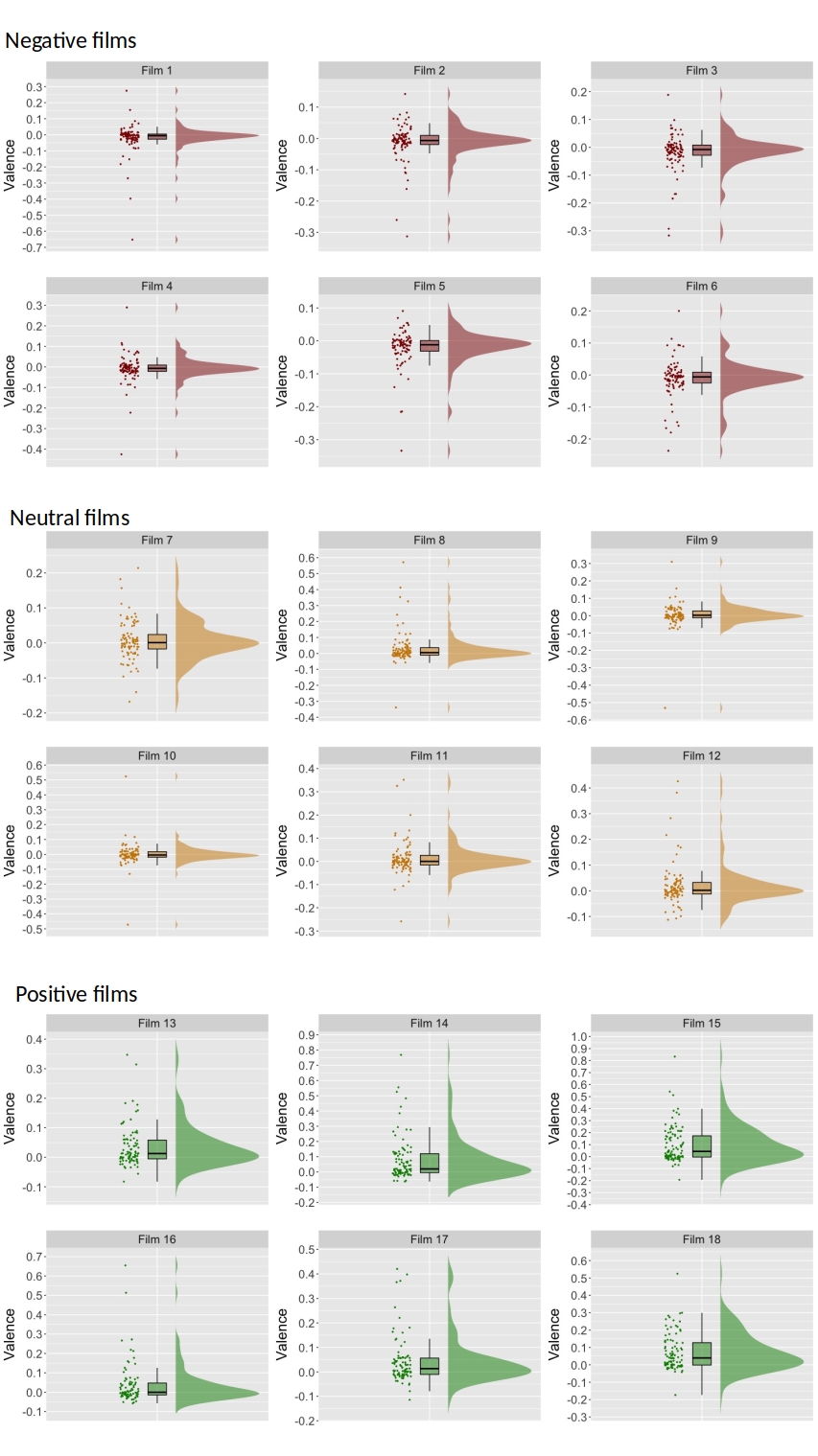

Supplement: Supplemental Information 4 — The dots represent individual data points. The box plot summarises minimum, first quartile, median, third quartile, and maximum. The half violin plots shows the density of the data. [file peerj-10-14160-s004.jpg]
