# Supplementary material for: Evaluation of film stimuli for the assessment of social-emotional processing: a pilot study
Source: PeerJ. 2022 Nov 23;10:e14160. doi: 10.7717/peerj.14160 (PMC9700451; doi:10.7717/peerj.14160)
Supplement: Supplemental Information 5 [file peerj-10-14160-s005.docx]

Supplemental Table S1: Films used in the present study

| Task | Film code | Film name | Link |
| --- | --- | --- | --- |
| Interpretation task | Film 1 | At Ease (3:11 – 4:36) | https://www.youtube.com/watch?v=1yXoxxrJzkQ |
|  | Film 2 | Fast and Loose (0:00 – 1:42) | https://www.youtube.com/watch?v=flTYcSzFp5o |
|  | Film 3 | Forget Me Not (8:50 – 10:09) | https://www.youtube.com/watch?v=U3q3LCu9u0w |
|  | Film 4 | F is for Friendship (0:13 – 1:45) | https://www.youtube.com/watch?v=5snCp929FQY |
|  | Film 5 | Lily (3:37 – 5:05) | https://www.youtube.com/watch?v=lxOaUJsNFKE |
|  | Film 6 | Pillars (6:12 – 7:39) | https://www.youtube.com/watch?v=h6rFoI5zC_I |
|  | Film 7 | Bare (3:58 – 5:52) | https://www.youtube.com/watch?v=TzwOTYHdI7Y |
|  | Film 8 | Company (0:26 – 2:08) | https://vimeo.com/306087436 |
|  | Film 9 | Pregnant Pause (0:41 – 2:14) | https://www.youtube.com/watch?v=xZK3QOX-580&t=655s |
|  | Film 10 | The Liberty (5:45 – 7:39) | https://www.youtube.com/watch?v=c8BmmFh_J88 |
|  | Film 11 | Whenever You’re Ready (1:47 – 3:30) | https://www.youtube.com/watch?v=mfkBDNu7jp4 |
|  | Film 12 | Youth, full (0:35 – 2:36) | https://www.youtube.com/watch?v=EZ78blhO24o |
|  | Film 13 | City Lights (3:57 – 5:12) | https://www.youtube.com/watch?v=Ub0DQvo2ZWw |
|  | Film 14 | Don’t Be a Hero (7:46 – 9:31) | https://www.youtube.com/watch?v=S7xzR2Fn02A |
|  | Film15 | Hello, Again (3:37 – 5:16) | https://www.youtube.com/watch?v=h21uXo1_Iz4 |
|  | Film 16 | Palm Trees and Power Lines (3:01 – 4:45) | https://www.youtube.com/watch?v=Na7PFgDySmw |
|  | Film 17 | The Proposal (0:10 – 1:49) | https://www.youtube.com/watch?v=RvqE23PSwIg |
|  | Film 18 | So It Goes (3:28 – 5:24) | https://www.youtube.com/watch?v=vqJfLseD4WA |
| Evoked emotions task | Film 1 | In a heartbeat (0:12 – 2:13) | https://www.youtube.com/watch?v=MxqGin4Pm-8 |
|  | Film 2 | Invisible Strings (1:57 – 2:30, 4:02 – 5:37) | https://www.youtube.com/watch?v=d92bzQTqwoM |
|  | Film 3 | Lifeboat (5:32 – 8:33) | https://www.youtube.com/watch?v=CdagKUkmATQ |
|  | Film 4 | Presentation (4:47 – 7:10) | https://www.youtube.com/watch?v=7Vq4Nf-zJoc&t=347s |
|  | Film 5 | Pride and Pack – Pride of Lions (17:58 – 20:09) | https://www.youtube.com/watch?v=kv94E6FZHLI |
|  | Film 6 | Work (0:18 – 2:22) | https://www.youtube.com/watch?v=nkqgjsMDYho |
|  | Film 7 | Finish Line (6:45 – 8:56) | https://www.youtube.com/watch?v=npsWa7XPFWQ&t=361s |
|  | Film 8 | K.I.T (3:41 – 5:43) | https://www.youtube.com/watch?v=8KuONLMdqe0 |
|  | Film 9 | Dreaming whilst Black (1:16 – 3:32) | https://www.youtube.com/watch?v=DuaR4qfAF8M |
|  | Film 10 | Ohio (5:36 – 7:47) | https://vimeo.com/87624949 |
|  | Film 11 | Reception (0:07 – 2:24) | https://www.youtube.com/watch?v=z9ncrJM4gd4 |
|  | Film 12 | RPG (0:00 – 2:10) | https://vimeo.com/170462812 |
|  | Film 13 | Blessing in Disguise (3:14 – 5:32) | https://vimeo.com/62920984 |
|  | Film 14 | Reality 2.0: Catcalling (0:07 – 2:50) | https://www.youtube.com/watch?v=cKDt7ZoYBI4 |
|  | Film 15 | Chinese Hi-Five | https://www.youtube.com/watch?v=dntuOC8Rl_c |
|  | Film 16 | Hot mess (0:24 – 2:49) | https://www.youtube.com/watch?v=Z845_DCLiFw |
|  | Film 17 | Russian Roulette (1:12 – 3:46) | https://www.youtube.com/watch?v=q5RcI8wfouU |
|  | Film 18 | Standby (0:21 – 2:26) | https://vimeo.com/207630463 |
