# Supplementary material for: Evaluation of film stimuli for the assessment of social-emotional processing: a pilot study
Source: PeerJ. 2022 Nov 23;10:e14160. doi: 10.7717/peerj.14160 (PMC9700451; doi:10.7717/peerj.14160)
Supplement: Supplemental Information 6 [file peerj-10-14160-s006.docx]

Supplemental Table S2. Valence of participants interpretations in laboratory and at home

| Location | Film category | Question 1 | Question 2 | X^2^(DF) statistic, p-value |
| --- | --- | --- | --- | --- |
|  |  | Valence  M (SD) | Valence  M (SD) |  |
| Laboratory | Neutral | -0.17 (0.49) | -0.05 (0.74) | Location: X^2^ (1) = 0.06, p = 0.806  Film category: X^2^ (2) = 873.00, p < 0.001  Question: X^2^ (1) = 56.70, p < 0.001  Film category x Question: $X^{2}$(2) = 3.80, p = 0.150  Film category x Location: X^2^ (2) = 1.22, p = 0.544  Question x Location: X^2^ (1) = 0.19, p = 0.664  Film category x Question X Location: X^2^ (2) = 3.38, 0.185 |
|  | Positive | 0.09 (0.62) | 0.24 (0.79) |  |
|  | Negative | -0.73 (0.49) | -0.49 (0.58) |  |
|  | Total | -0.27 (0.65) | -0.10 (0.77) |  |
| Home | Neutral | -0.19 (0.59) | -0.07 (0.79) |  |
|  | Positive | 0.09 (0.65) | 0.30 (0.80) |  |
|  | Negative | -0.64 (0.58) | -0.51 (0.67) |  |
|  | Total | -0.24 (0.68) | -0.09 (0.83) |  |
