# Supplementary material for: Evaluation of film stimuli for the assessment of social-emotional processing: a pilot study
Source: PeerJ. 2022 Nov 23;10:e14160. doi: 10.7717/peerj.14160 (PMC9700451; doi:10.7717/peerj.14160)
Supplement: Supplemental Information 7 [file peerj-10-14160-s007.docx]

Supplemental Table S3. Impact of location on valence of facial expressions

| Location | Film category | Facial valence  M (SD) | F (DF) statistic, p-value |
| --- | --- | --- | --- |
| Laboratory | Neutral | 0.02 (0.07) | Location: F(1,108.08) = 0.84, p = 0.363  Film category: F(2,1581.80) = 108.56, p < 0.001  Film category x Location: F(2,1581.80) = 0.34, p = 0.712 |
|  | Positive | 0.07 (0.12) |  |
|  | Negative | -0.01 (0.06) |  |
| Home | Neutral | 0.01 (0.08) |  |
|  | Positive | 0.06 (0.12) |  |
|  | Negative | -0.02 (0.07) |  |
