# Supplementary material for: Evaluation of film stimuli for the assessment of social-emotional processing: a pilot study
Source: PeerJ. 2022 Nov 23;10:e14160. doi: 10.7717/peerj.14160 (PMC9700451; doi:10.7717/peerj.14160)
Supplement: Supplemental Information 8 [file peerj-10-14160-s008.docx]

Supplemental Table S4. Impact of location on self-reported mood ratings

| Location | Film category | Mood rating  M (SD) | F (DF) statistic, p-value |
| --- | --- | --- | --- |
| Laboratory | Neutral | 0.13 (4.06) | Location: F(1,118.17) = 1.64, p = 0.203  Film category: F(2,2026.67) = 861.38, p < 0.001  Film category x Location: F(2,2026.67) = 0.31, p = 0.731 |
|  | Positive | 3.95 (4.38) |  |
|  | Negative | -5.30 (3.65) |  |
| Home | Neutral | 0.36 (3.40) |  |
|  | Positive | 4.34 (3.82) |  |
|  | Negative | -4.72 (3.31) |  |
