# Supplementary material for: Evaluation of film stimuli for the assessment of social-emotional processing: a pilot study
Source: PeerJ. 2022 Nov 23;10:e14160. doi: 10.7717/peerj.14160 (PMC9700451; doi:10.7717/peerj.14160)
Supplement: Supplemental Information 9 — EDEQ = Eating Disorders Examination Questionnaire; HADS = Hospital Anxiety and Depression Scale; BES = Beliefs about Emotions Scale. The upper diagonal shows correlations with the mean valence of participants responses to question 1 and the lower diagonal shows correlations with the mean valence of participants responses to question 2. [file peerj-10-14160-s009.docx]

Supplemental Table S5: Correlation matrix of self-report variables and valence of written responses to question 1 (upper diagonal) and question 2 (lower diagonal)

|  | Mean valence | EDEQ total | HADS anxiety | HADS depression | BES total |
| --- | --- | --- | --- | --- | --- |
| Mean valence |  | τ= 0.01, p > 0.99 | τ= 0.02, p > 0.99 | τ= 0.001, p > 0.99 | τ= 0.07, p > 0.99 |
| EDEQ total | τ= 0.04, p > 0.99 | 1 | τ= 0.30, p < 0.001 | τ= 0.18, p = 0.39 | τ= 0.14, p = 0.17 |
| HADS anxiety | τ= 0.02, p > 0.99 | τ= 0.30, p = 0.01 | 1 | τ= 0.31, p = 0.01 | τ= 0.17, p = 0.54 |
| HADS depression | τ= 0.04, p > 0.99 | τ= 0.18, p = 0.39 | τ= 0.31, p = 0.01 | 1 | τ= 0.13, p = 0.85 |
| BES total | τ=0.08, p > 0.99 | τ= 0.14, p = 0.17 | τ= 0.17, p = 0.54 | τ= 0.13, p = 0.85 | 1 |

EDEQ = Eating Disorders Examination Questionnaire; HADS = Hospital Anxiety and Depression Scale; BES = Beliefs about Emotions Scale. The upper diagonal shows correlations with the mean valence of participants responses to question 1 and the lower diagonal shows correlations with the mean valence of participants responses to question 2.
