# Supplementary material for: Evaluation of film stimuli for the assessment of social-emotional processing: a pilot study
Source: PeerJ. 2022 Nov 23;10:e14160. doi: 10.7717/peerj.14160 (PMC9700451; doi:10.7717/peerj.14160)
Supplement: Supplemental Information 10 — EDEQ = Eating Disorders Examination Questionnaire; HADS = Hospital Anxiety and Depression Scale; BES = Beliefs about Emotions Scale. The upper diagonal shows correlations with the participants’ mean mood ratings and the lower diagonal shows correlations with the participants’ mean valence of their facial affect [file peerj-10-14160-s010.docx]

Supplemental Table S6: Correlation matrix of self-report variables and mean mood ratings (upper diagonal) and mean valence of facial affect (lower diagonal)

|  | Mean valence of facial affect | EDEQ total | HADS anxiety | HADS depression | BES total |
| --- | --- | --- | --- | --- | --- |
| Mean mood | - | τ= -0.04, p > 0.99 | τ= -0.08, p > 0.99 | τ= -0.11, p = 0.95 | τ< 0.001, p > 0.99 |
| EDEQ total | τ= -0.05, p > 0.99 | 1 | τ= 0.33, p < 0.001 | τ= 0.17, p = 0.55 | τ= 0.16, p = 0.55 |
| HADS anxiety | τ= 0.027 p > 0.99 | τ= 0.33, p < 0.001 | 1 | τ= 0.30, p = 0.01 | τ= 0.20, p = 0.31 |
| HADS depression | τ= -0.07, p > 0.99 | τ= 0.17, p = 0.55 | τ= 0.30, p = 0.01 | 1 | τ= 0.17, p = 0.55 |
| BES total | τ= -0.07, p > 0.99 | τ= 0.16, p = 0.55 | τ= 0.20, p = 0.31 | τ= 0.17, p = 0.55 | 1 |

EDEQ = Eating Disorders Examination Questionnaire; HADS = Hospital Anxiety and Depression Scale; BES = Beliefs about Emotions Scale. The upper diagonal shows correlations with the participants’ mean mood ratings and the lower diagonal shows correlations with the participants’ mean valence of their facial affect
